# Analysis of the COVID-19 Epidemic Transmission Network in Mainland China: K-Core Decomposition Study

**DOI:** 10.2196/24291

**Published:** 2020-11-13

**Authors:** Lei Qin, Yidan Wang, Qiang Sun, Xiaomei Zhang, Ben-Chang Shia, Chengcheng Liu

**Affiliations:** 1 School of Statistics University of International Business and Economics Beijing China; 2 Graduate Institute of Business Administration College of Management Fu Jen Catholic University New Taipei City Taiwan; 3 School of Statistics Capital University of Economics and Business Beijing China

**Keywords:** COVID-19, epidemic network, prevention and control, k-core decomposition

## Abstract

**Background:**

Since the outbreak of COVID-19 in December 2019 in Wuhan, Hubei Province, China, frequent interregional contacts and the high rate of infection spread have catalyzed the formation of an epidemic network.

**Objective:**

The aim of this study was to identify influential nodes and highlight the hidden structural properties of the COVID-19 epidemic network, which we believe is central to prevention and control of the epidemic.

**Methods:**

We first constructed a network of the COVID-19 epidemic among 31 provinces in mainland China; after some basic characteristics were revealed by the degree distribution, the k-core decomposition method was employed to provide static and dynamic evidence to determine the influential nodes and hierarchical structure. We then exhibited the influence power of the above nodes and the evolution of this power.

**Results:**

Only a small fraction of the provinces studied showed relatively strong outward or inward epidemic transmission effects. The three provinces of Hubei, Beijing, and Guangzhou showed the highest out-degrees, and the three highest in-degrees were observed for the provinces of Beijing, Henan, and Liaoning. In terms of the hierarchical structure of the COVID-19 epidemic network over the whole period, more than half of the 31 provinces were located in the innermost core. Considering the correlation of the characteristics and coreness of each province, we identified some significant negative and positive factors. Specific to the dynamic transmission process of the COVID-19 epidemic, three provinces of Anhui, Beijing, and Guangdong always showed the highest coreness from the third to the sixth week; meanwhile, Hubei Province maintained the highest coreness until the fifth week and then suddenly dropped to the lowest in the sixth week. We also found that the out-strengths of the innermost nodes were greater than their in-strengths before January 27, 2020, at which point a reversal occurred.

**Conclusions:**

Increasing our understanding of how epidemic networks form and function may help reduce the damaging effects of COVID-19 in China as well as in other countries and territories worldwide.

## Introduction

In December 2019, several cases of pneumonia of unknown etiology were detected in Wuhan City, Hubei Province, China. Chinese authorities identified the causative agent as a novel coronavirus of probable bat origin [1], and the World Health Organization (WHO) officially named the disease COVID-19 on February 11, 2020 [2]. Compared with the outbreak of severe acute respiratory syndrome (SARS) in China in 2003, COVID-19 has spread faster and infected more people [3]; furthermore, it is more difficult to prevent and control. Considering that the number of cases started increasing exponentially, the Chinese government imposed a lockdown in Wuhan on January 23, 2020, aiming to cut off the route of virus transmission through a traffic blockade [4]. After that, COVID-19 was clearly brought under control. Since March 2020, this ongoing epidemic has now spread to more than 200 countries and territories, and it is undoubtedly casting a shadow over the global economy. To mitigate the impact of epidemics and ensure the continuity of global social development, an exploration of the influential nodes and structural properties of the COVID-19 epidemic network is urgently needed.

There have been extensive studies on epidemiological transmission mechanisms from diverse perspectives, such as epidemiology, medical statistics, spatial information science, sociology, and dynamic models [5-10]. Due to the wide spread, epidemic data are often presented in the form of a network. The application advantages of complex network theory are gradually being highlighted [11,12]. In the framework of complex network theory, k-core decomposition is usually considered for identifying influential spreaders [13,14] and finding specific structural information [15-18].

K-core decomposition is a well-established method for analyzing the structures of large-scale graphs [19,20]. The original idea of k-core decomposition can be traced back to the concepts of coloring number [21] and degeneracy [22], and the commonly accepted concept was first proposed by Seidman [19]. Further studies mainly involved two aspects. One focuses on solving the theoretical problem of the k-core pruning process in different networks, and the other involves finding the densest part of the network by k-core decomposition across a broad range of scientific subjects, including biology, ecology, computer science, social networks, information spreading, and community detection.

In particular, k-core decomposition provides a method for identifying hierarchies in a network. It considers the coreness of nodes by dividing networks into layers or shells. Compared with other methods, k-core decomposition possesses a significant advantage of computational simplicity [23]. It has found a number of applications as a means to understand the importance of nodes within large-scale network structures [20].

While the existing literature is replete with explorations of epidemic networks and applications of k-core decomposition, few studies involve the effective combination of the two. In the context of the COVID-19 epidemic in mainland China, this paper applies k-core decomposition to the structural analysis of the network of the epidemic with the purpose of arriving at some novel conclusions to aid the prevention and control of the disease. Our contribution is threefold. First, the COVID-19 epidemic data of all provinces in mainland China are timely and unique. Second, we obtained the related static and dynamic conclusions of influential nodes and the hierarchical structure by applying k-core decomposition to the COVID-19 epidemic network; furthermore, we detected the common characteristics of provinces represented by these important nodes. Finally, the influence power of k-shell nodes and the evolution of this power, measured by out-strength and in-strength, can promote our understanding of the roles of the provinces in epidemic transmission.

In this paper, we briefly introduce the construction and further analytical methods of the COVID-19 epidemic network after describing the data used. We then summarize some basic structural properties of the epidemic network by means of degree distribution. Then, k-core decomposition is applied to the constructed whole period and daily networks to statically and dynamically investigate the network structure. Finally, the influence power (outgoing and incoming) of the k-shell and its evolution are exhibited.

## Methods

### Data

In view of the construction of COVID-19 epidemic network among the provinces in mainland China, the cross-provincial traveling data (1690 observations) of confirmed patients were considered. In our study, the traveling extent mainly focused on 31 provinces (all except Taiwan, Hong Kong, and Macao) in China, and the traveling options were restricted to air and train travel. The aforementioned data were obtained from a website [24], that includes records from confirmed official WeChat and Weibo accounts as well as official websites. In addition, some unverifiable data were eliminated. In total, 1615 observations (328 observations by plane and 1287 observations by train) were retained, and the period of the observations ranged from December 27, 2019, to February 25, 2020, covering 61 days. It should be noted that a connection between province A and province B can be established in the COVID-19 epidemic network if the traveling data show that a latent confirmed patient traveled from province A to province B and was diagnosed in province B.

Some variables at the provincial level, such as gross domestic product (GDP) per capita, population, volume of passenger transport, starting date of first-level response to the major public health emergency, response time, and distance from Hubei Province were also used in the follow-up study on the common characteristics of provinces represented by important nodes. Specifically, the GDP, population, volume of passenger transport, and starting date of first-level response to the major public health emergency were obtained from the National Bureau of Statistics and Provincial Health Committees. Response time was calculated by the average number of days between the arrival date and the confirmed date, and for the spatial distances from Hubei Province, we referred to Yu [25].

### Methodology

Considering that the causative agent of COVID-19 is carried by humans—in other words, it is mainly spread by human-to-human transmission—the cross-provincial traveling records of confirmed patients can directly depict the epidemic network to a large extent. Specific to the COVID-19 epidemic network *G =* (*V*,*E*), province A and province B can be viewed as node *V_A_* and node *V_B_*, respectively; there are corresponding directional edges *E_AB_* or *E_BA_* between nodes *V_A_* and *V_B_* if a confirmed patient traveled from province A to province B or from province B to province A. On this basis, the directionality information of edges was well considered in the following analyses of degree distribution and influence power, but was not considered in the k-core decomposition.

As mentioned before, three methods were applied to the further structural analysis of the COVID-19 epidemic network: degree distribution, k-core decomposition, and influence power. Under the method framework of degree distribution, the degree of a node is defined as its number of connected edges, and it can be divided into an out-degree and in-degree according to the direction of the edges. In addition, the cumulative degree distribution represents the probability distribution of nodes with degree not less than *k*. In the Degree Distribution section, we will show the cumulative distributions of both the out-degrees and in-degrees to reveal some basic characteristics of the epidemic network. The degree distribution provides useful information about the network; however, it is limited by the revelation of the complete structure. Therefore, other network methods should be applied, such as k-core decomposition.

**Figure 1:**
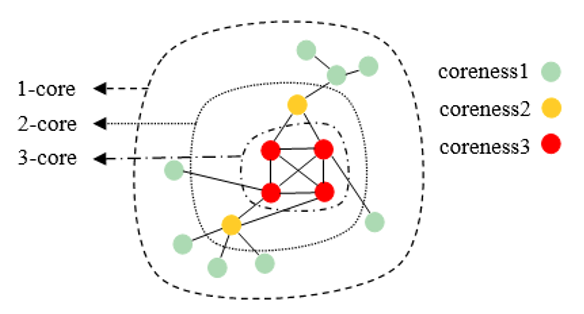
Illustration of the k-core decomposition of a small network. The sets of nodes belonging to the 1-core, 2-core, and 3-core are enclosed by different types of lines. The different k-shells can also be distinguished by the colors of the nodes.

The advantage of k-core decomposition is that it can be used to detect the core and surrounding shell of a complex network. The fundamental application of this method is to decompose the network into multiple partitions, which is a straightforward procedure. Let *G=*(*V,E*) be a graph with *n* = |*V*| nodes and *e* = |*E*| edges. The so-called k-core is a maximal connected subgraph of *G* in which the degree of all nodes is at least *k*. A node *V_i_* has coreness *ks*(*V_i_*)=*k* if it belongs to the k-core instead of the (k+1)-core. We note that the value of *k* is automatically learned from the observed network data and is also independent of our prior anticipation. More specifically, the k-core decomposition method can realize the k-shell classification of all nodes of *G* by removing them iteratively, as follows. First, we removed all nodes with degree *k*=1 and assigned the coreness value *ks*=1 to the removed nodes. Second, a pruning process was repeated until only nodes with degree *k*>1 remained. Next, we performed a similar pruning process for the nodes with degree *k*=2 and assigned the corresponding coreness value *ks*=2. The above procedures were repeated until all nodes of *G* were removed and assigned to one of the k-shells. Figure 1 illustrates the simple k-core decomposition of a connected graph.

To identify the important nodes of the COVID-19 epidemic network and reveal its hierarchical structure through k-core decomposition, a geospatial network topology map of the whole time period showing the coreness of each node (province) and their connections was plotted. From the dynamic angle, we also focused on the daily or weekly evolution of *k*_max_ as well as the number of nodes and edges. In addition, a group of scatter charts was drawn to describe the relationship between the coreness and characteristics of the provinces to demonstrate the common characteristics of important provinces in COVID-19 epidemic transmission. Most importantly, we could clearly present the hierarchical structure of the epidemic network by week. Finally, we introduced the method of influence power to measure the transmission effects (outgoing and incoming) among provinces.

## Results

### Degree Distribution

The method of degree distribution can offer a glimpse of the properties of a network. In the study of a network, the degree *k* of a node, which is regarded as the number of its direct neighbors, can be measured by the number of connections with other nodes. Hence, the degree distribution *P*(*k*) relates to the probability that a randomly chosen node has *k* connections. Considering the directivity, the out-degree and in-degree are the respective numbers of outgoing and incoming connections. Correspondingly, the probability that a randomly chosen node has out-degree *k_out_* and in-degree *k_in_* are represented by *P*(*k_out_*) and *P*(*k_in_*).

**Figure 2:**
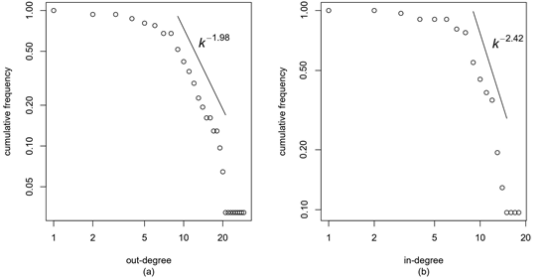
Cumulative frequency graphs of the out-degrees and in-degrees. (a) The cumulative distribution of the out-degrees follows an approximate power law with exponent γ=1.98. (b) The cumulative distribution of the in-degrees follows an approximate power law with exponent γ=2.42. Both axes are in logarithmic scale.

In terms of the COVID-19 epidemic network, where links are directed among 31 provinces, the cumulative out-degree and in-degree distributions are shown in Figure 2. We note that the cumulative frequency of the out-degrees (in-degrees) is the proportion of nodes in which the out-degree (in-degree) is not less than *k*. We can clearly see that they all follow the power law *P*(*k*)~*k^-γ^*, and the values of the exponent *γ* are 1.98 and 2.42 for the cumulative distributions of the out-degrees and in-degrees, respectively. The above power-law distributions demonstrate that most provinces have low out-degrees, and only a small fraction of the provinces maintain relatively strong outward epidemic transmission effects on other provinces. Similarly, the overwhelming majority of provinces have low in-degrees, and the proportion of provinces with stronger inward epidemic transmission effects is small. This finding is consistent with the fact that a few provinces, such as Hubei, Beijing, and Henan, were seriously affected by the COVID-19 epidemic, while others were less affected.

**Figure 3:**
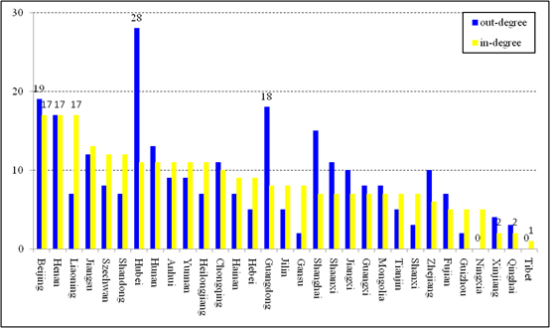
Histogram describing the specific out-degrees and in-degrees of the 31 Chinese provinces in the COVID-19 epidemic network.

**Figure 4:**
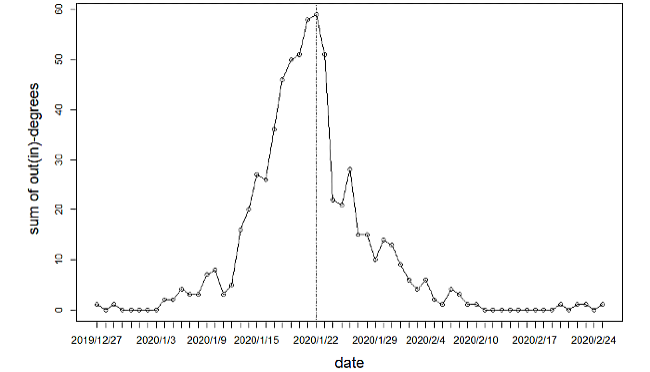
Evolution of the sum of the out-degrees (in-degrees) in the COVID-19 epidemic network for each day.

To identify the transmission role of each province in the COVID-19 epidemic, a histogram describing the specific out-degrees and in-degrees of the 31 studied provinces is shown in Figure 3. In terms of the out-degree, the corresponding values of seven provinces (Xinjiang, Shanxi, Qinghai, Gansu, Guizhou, Ningxia, and Tibet) are less than 5; furthermore, the values for the provinces of Ningxia and Tibet are 0. The reason that few patients came from these provinces may be their location in the western part of mainland China, which has a relatively recessive economy and a less transient population. The provinces of Hubei, Beijing, and Guangzhou show the three highest out-degrees of 28, 19, and 18, respectively. Wuhan, the capital city of Hubei Province, was the first place to witness confirmed patients and is the epicenter of the epidemic outbreak. As two of the most developed provinces in mainland China, Beijing and Guangzhou had significant impacts on other provinces due to their larger transient populations. In the case of in-degree, three provinces (Xinjiang, Qinghai, and Tibet) have in-degrees <5, and the three provinces with the highest values are Beijing, Henan, and Liaoning (all 17). Similarly, provinces with higher population mobility are more significantly affected than isolated provinces. The higher in-degrees of Henan and Liaoning may be caused by the return of confirmed migrant laborers during the Spring Festival [26]. We also attempted to characterize the intuitive time attributes of the COVID-19 epidemic network, and we presented the evolution of the sum of the out-degrees (in-degrees) of the daily networks in Figure 4. The traveling route of a confirmed patient usually involves two different provinces as departure and destination, which means that one patient outbound relates to one inbound patient. Thus, the out-degrees of all nodes in a network are equal to its in-degrees. The sum of the out-degrees (in-degrees) shows an inverted U-shape, and the maximum was achieved on January 22, 2020. It can be seen that the complexity of COVID-19 epidemic network is time-varying, and it stood out on January 22, 2020.

After grasping some basic characteristics of the epidemic network, it is reasonable to assume that small groups of nodes organize in a hierarchical manner into increasingly large groups. However, the method of degree distribution lacks cognition of which node belongs to which layer, and the differences between layers are not sufficiently clear. K-core decomposition, which disentangles the hierarchical structure of networks by progressively focusing on their central cores, is of great use in obtaining the above detailed structural information.

### K-Core Decomposition

**Figure 5:**
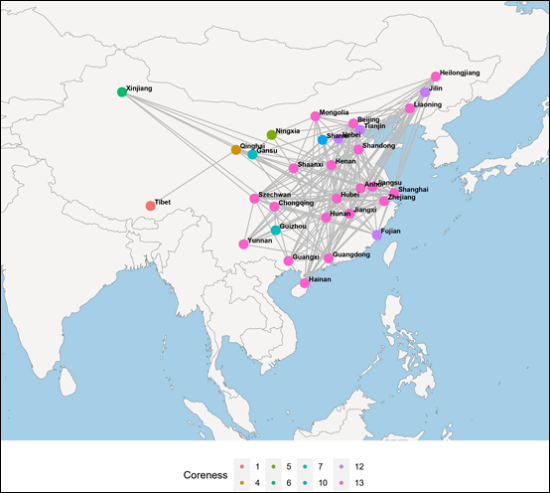
Topological image in geospatial space depicting the network relationship and the coreness of each node over the whole period. The color of the node represents its coreness, which corresponds to the k-shell it belongs to.

**Figure 6:**
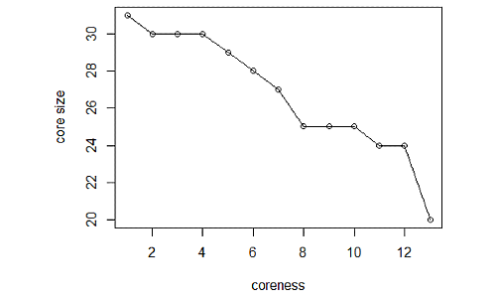
Plot of the core size versus the coreness of the network nodes over the whole study period. The core size represented in the vertical axis indicates the number of nodes with corenesses greater than or equal to the corresponding coreness on the horizontal axis.

The generally accepted concept of k-core decomposition focuses more on the connections between nodes, while directionality can sometimes be ignored [16,18]. Specific to our COVID-19 network analysis, directionality was not taken into account in the application of k-core decomposition. Statically, the network relationship and the coreness of each node over the whole period (from December 27, 2019, to February 25, 2020) are displayed in Figure 5. Here, it should be pointed out that a visualization software called Gephi [27] was used to exhibit the topological image in geospatial space to provide clear insight into the exact location of each node. It can be seen that there are 20 nodes with the highest coreness 13, accounting for 64.52% of 31 provinces, while the lowest coreness of 1 appears for the remote province of Tibet. More specifically, all provinces adjacent to the outbreak area (Hubei Province) have the highest coreness values. In addition, Figure 6 plots the core size with respect to the coreness over the whole period. It can be seen that increasing coreness usually results in shrinking of the network. Combining Figure 5 with Figure 6, we can see that 25 nodes have corenesses >8, and the nodes in the four innermost layers account for 80.65% of the entire epidemic network. These results further indicate that the COVID-19 epidemic affected the overwhelming majority of provinces in mainland China.

**Figure 7:**
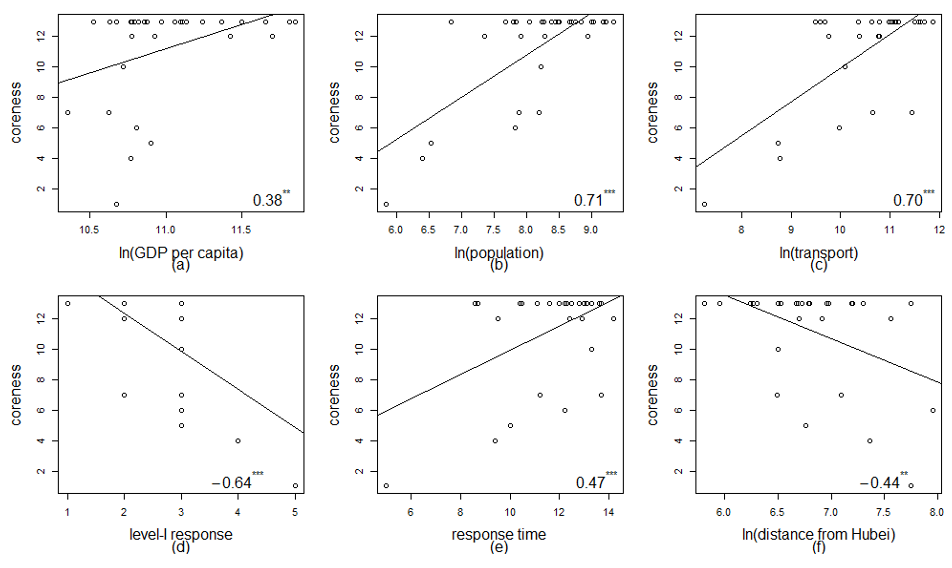
Correlation diagrams of the corenesses and characteristics of the provinces. The horizontal and vertical axes denote the characteristic variables and corenesses, respectively. The numbers in the lower right corner of each subgraph are the corresponding correlation coefficient. **5% significance, ***1% significance.

To investigate which characteristics of the provinces are the key factors determining the coreness of each node, six variables (GDP per capita, population, volume of passenger transport, starting date of first-level response to major public health emergency, response time, and distance from Hubei Province) at the provincial level were introduced to draw a correlation diagram, as shown in Figure 7. Here, a logarithmic transformation was applied to the indicators of GDP per capita, population, volume of passenger transport, and distance from Hubei Province. It can be deduced from Figure 7 that the starting date of the first-level response to the major public health emergency and the distance from Hubei Province are significant negative correlation factors, while the other factors tend to be positively correlated. For example, we can reasonably assume that the starting date of the first-level response to the major public health emergency is related to the severity of the COVID-19 epidemic. The earlier the first-level response, the stronger the infection spread in that province. Generally, people carrying SARS-CoV-2 are more likely to be found in provinces with larger volumes of passenger transport, which also determines the importance of these provinces to the spread of the epidemic. These findings echo the analysis of the degree distribution to some extent.

**Figure 8:**
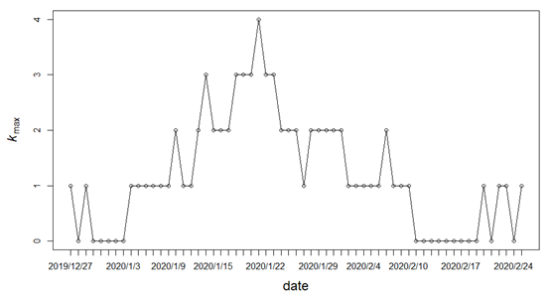
Daily evolution of the maximum coreness (*k_max_*) in the COVID-19 epidemic network.

In fact, the COVID-19 epidemic network is more likely to be dynamic than fixed over a period of time, and it is necessary to investigate its time-varying maximum coreness *k*_max_. Based on this, we first constructed daily epidemic networks, and Figure 8 presents the evolution of *k*_max_. The figure shows that there is an obvious trend of initial rising and then falling for *k*_max_, and the maximum peak appears on January 21, 2020. We can conclude that the epidemic network from the end of January to the beginning of February is relatively complex. Considering the simple structure of the daily epidemic networks, the experiential patterns of summarized epidemic transmission are limited. Furthermore, the whole period can be divided into intervals, with a fixed window of 7 days.

**Figure 9:**
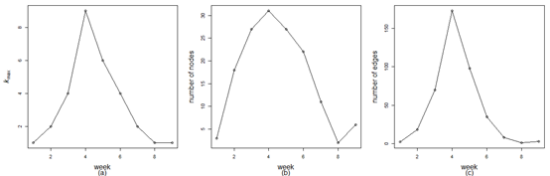
Evolutions of the maximum coreness (*k_max_*), number of nodes, and number of edges in the weekly COVID-19 epidemic networks.

Similar to Figure 8, Figure 9 shows the weekly evolutions of the maximum coreness *k*_max_, node number, and edge number. In terms of their common trends, there is a significant increase before the fourth week, at which point a steep decline appears; thus, the fourth week (January 17 to January 23, 2020) becomes its peak point. Considering the differences among the three, the number of nodes and edges goes up slightly after the eighth week. The above findings again confirm that the fourth week is the critical period of the COVID-19 epidemic outbreak, and the network structure formed in the surrounding weeks is relatively complex. The above statistical results depict the dynamic development of the COVID-19 epidemic. The outbreak and spread of COVID-19 led to a surge in the number of confirmed cases. The overwhelming majority of provinces started their first-level responses to the major public health emergency from January 23 to January 25, 2020, and many measures, such as isolating at home and wearing masks, were taken to contain the outbreak. The following swift drop in the number of confirmed cases demonstrates the effectiveness of these measures.

**Figure 10:**
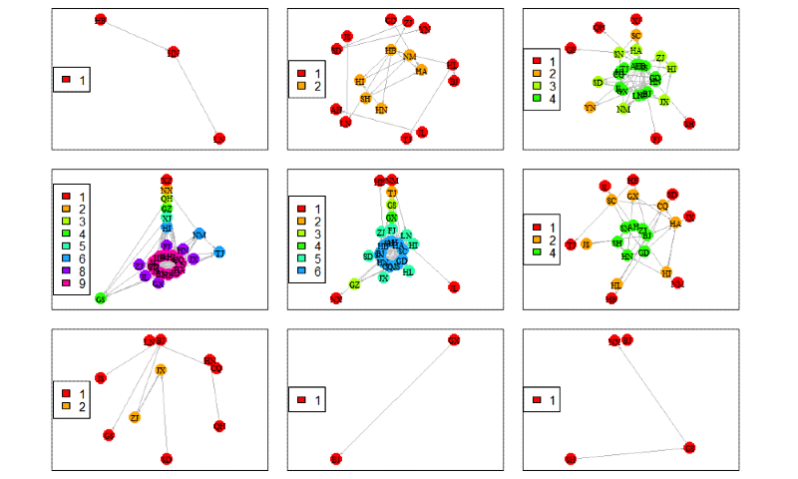
Dynamic networks of the COVID-19 epidemic showing the node composition and hierarchical structure. The color and label of each node denote the coreness and geographical province, respectively. The subgraphs from top to bottom and from left to right correspond to the nine weeks in order in the period from December 27, 2019 to February 25, 2020. AH: Anhui; BJ: Beijing; CQ: Chongqing; FJ: Fujian; GD: Guangdong; GS: Gansu; GX: Guangxi; GZ: Guizhou; HA: Henan; HB: Hubei; HE: Hebei; HI: Hainan; HL: Heilongjiang; HN: Hunan: JL: Jilin; JS: Jiangsu; JX: Jiangxi; LN: Liaoning; NM: Mongolia; NX: Ningxia; QH: Qinghai; SC: Szechwan; SD: Shandong; SH: Shanghai: SN: Shaanxi; SX: Shanxi; TJ: Tianjin; XJ: Xinjiang; XZ: Tibet; YN: Yunnan; ZJ: Zhejiang.

To visualize the epidemic transmission process dynamically, Figure 10 shows the node composition and hierarchical structure of the COVID-19 epidemic network by week. In general, the epidemic network structure corresponding to the third to sixth weeks tends to be more complex, and the fourth week stands out. Chinese spring rush may give a reasonable explanation. Taking the period from the third to sixth week as an example, there is no doubt that Tibet has kept the lowest coreness all the way, while provinces of Anhui, Beijing and Guangdong show the highest coreness. There is a novel discovery about the changes of coreness in the epidemic outbreak area Hubei Province, which kept the highest level from the third to fifth week, and suddenly dropped to the lowest in the sixth week. To some extent, the two-week-lagging effective control of COVID-19 epidemic transmission by lockdown measures imposed by Wuhan government on January 23, 2020 is verified.

### Influence Power

Epidemics generally occur in some regions first and then spread out rapidly when the situation in these regions becomes more serious and cannot be controlled. Specific to the network structure of an epidemic, the so-called outbreak or serious areas have high centrality and occupy a certain shell. Naturally, after applying k-core decomposition to the COVID-19 epidemic network in a fixed period, the extent to which each k-shell will influence the other shells becomes a more attractive question. Here, the method of influence power can provide a good answer. Under the framework of influence power, there are two indicators, out-strength and in-strength, that can respectively evaluate the power of each node to influence others and be influenced by others. Correspondingly, the calculation depends on the number of outgoing and incoming links. In terms of the COVID-19 epidemic network, if a large number of outgoing routes appear in one province, this province usually has a great transmission influence on other provinces. Similarly, if one province has a large number of incoming routes, it will also be greatly influenced by other provinces. Hence, the following formula can be used to further quantify the influence power *η_k_* of the k-shell:

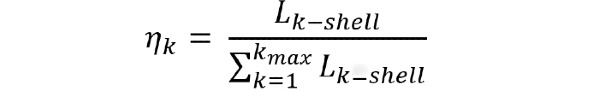



where *η_k_* is the calculated ratio of the influence power and *L_k-shell_* relates to the number of outgoing (incoming) links in each k-shell of concern.

The results of influence power (out-strength and in-strength) of each shell and the innermost nodes based on the COVID-19 epidemic network in the whole period are shown in Table 1 and Table 2, respectively. The maximum coreness of the COVID-19 epidemic network is 13, and almost all the outgoing links (1164/1220, 95.41%) are from the provinces with the highest coreness. Among these provinces, more than half of the outgoing links (689/1220, 56.48%) come from Hubei Province, which is the origin of the COVID-19 epidemic. The provinces of Guangdong and Beijing rank second and third, accounting for 4.51% (55/1220) and 3.77% (46/1220), respectively. Hainan Province, which is located in the same shell as Guangdong and Beijing, has the lowest proportion of outgoing links (7/1220, 0.57%). We can conclude that it is important to identify the key “outgoing” provinces for the prevention and control of the COVID-19 epidemic.

Furthermore, Table 2 can help us understand the “incoming” role of each shell and the innermost nodes in the transmission process of the COVID-19 epidemic. Consistent with the findings in Figure 5, the epidemic network in the whole period can be pruned recursively into 8 shells. In addition, there are nodes with incoming links in each shell. The highest proportion of incoming links, up to 86.80% (1059/1220), appears in the provinces with the highest corenesses. Among these provinces, the top three are Henan (152/1220, 12.46%), Szechwan (82/1220, 6.72%), and Guangxi (80/1220, 6.56%), while Zhejiang (16/1220, 1.31%) has the lowest proportion. Focusing on the breakout area of Hubei Province, there are only 28 incoming links (28/1220, 2.30%), in contrast to its largest number of outgoing links; this indicates that this province is less influenced by other provinces. On the whole, the in-strength performance of the provinces is different from the out-strength, and the “incoming” roles of these provinces tend to be more equal.

**Table 1:** Comparison of the out-strengths of each shell (coreness) and the innermost nodes (provinces) based on the COVID-19 epidemic network in the study period (N=1246 links), n (%).

Characteristic		Incoming links
**Coreness**		
	4	3 (0.25)
	6	5 (0.41)
	7	6 (0.49)
	10	3 (0.25)
	12	39 (3.20)
	13	1164 (95.41)
**Province^a^**		
	Hainan	7 (0.57)
	Henan	33 (2.70)
	Shaanxi	38 (3.11)
	Hunan	42 (3.44)
	Beijing	46 (3.77)
	Guangdong	55 (4.51)
	Hubei	689 (56.48)

^a^Because some nodes with 2 shells (Ningxia and Tibet) do not have outgoing links, only 6 shells of the COVID-19 epidemic network in the whole period are included in the table.

**Table 2:** Comparison of the in-strengths of each shell (coreness) and the innermost nodes (provinces) based on the COVID-19 epidemic network in the study period (N=1220 links), n (%).

Characteristic		Incoming links
**Coreness**		
	1	1 (0.08)
	4	5 (0.41)
	5	5 (0.41)
	6	2 (0.16)
	7	27 (2.21)
	10	22 (1.80)
	12	99 (8.11)
	13	1059 (86.80)
**Province**		
	Zhejiang	16 (1.31)
	Hubei	28 (2.30)
	Liaoning	58 (4.75)
	Jiangsu	63 (5.16)
	Hunan	71 (5.82)
	Guangxi	80 (6.56)
	Szechwan	82 (6.72)
	Henan	152 (12.46)

**Figure 11:**
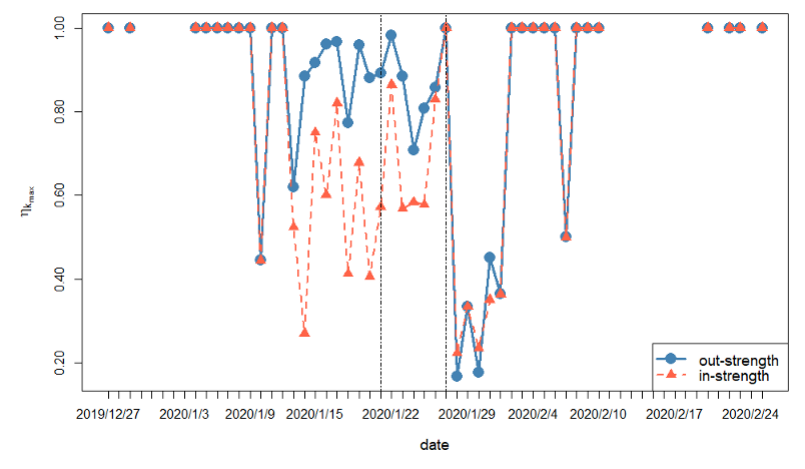
Daily evolution of the innermost shell from the perspectives of out-strength and in-strength in the COVID-19 epidemic network. The vertical dotted lines correspond to January 21, 2020, and January 27, 2020, respectively.

In some cases, the dynamic influence of a shell consisting of the most important nodes in the whole network is more worthy of attention. The above influence can also be measured by the method of influence power, which depends on the time-varying set of the innermost nodes and all the related directional links. On the basis of the daily COVID-19 epidemic networks, Figure 11 presents the evolution of 
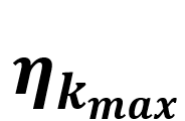
 from the perspectives of out-strength and in-strength, which elucidates the dynamic “outgoing” and “incoming” roles of those important provinces. It is notable that a relatively large gap between out-strength and in-strength exists on January 21, 2020, which echoes the most complex daily network indicated in Figure 8. We also observed that the out-strengths of the innermost nodes are larger than the in-strengths before January 27, 2020, which indicates that those provinces tend to have more influence on others rather than being influenced by others. After that, due to the stricter control measures imposed by those provinces, their out-strengths and the above contrast greatly weaken. To a certain extent, the above findings confirm the effectiveness of the control measures implemented by the Chinese government during the COVID-19 epidemic.

## Discussion

### Principal Findings

In economics, epidemiology, and many other fields, the increasing number of participants and volume of data further complicate the formation of networks. It is critical to extract effective information from these large and complex networks. Hence, it is necessary to identify and focus on the central nodes that drive the whole network instead of paying the same amount of attention to all the nodes. As mentioned above, degree distribution is the simplest way to measure the centrality of each node in a network; it only involves the local structure around the node. Specifically, in a binary network, the degree distribution depends on the number of edges of the considered node. In a directed network, the connecting edges of a node may have two directions, outgoing and incoming, which correspond to the out-degree and in-degree under the framework of degree distribution. Furthermore, the concept of degree has generally been extended to the sum of weights when analyzing a weighted directed network [28], and the strength (out-strength and in-strength) of nodes has been proposed. Additionally, to acquire more detailed information about a network structure, k-core decomposition can be employed to disentangle the hierarchical structure of the network by progressively focusing on its central core. In summary, the indicators of degree, coreness, and strength we adopted above can provide different perspectives to understand the nodes and structures of a network.

**Figure 12:**
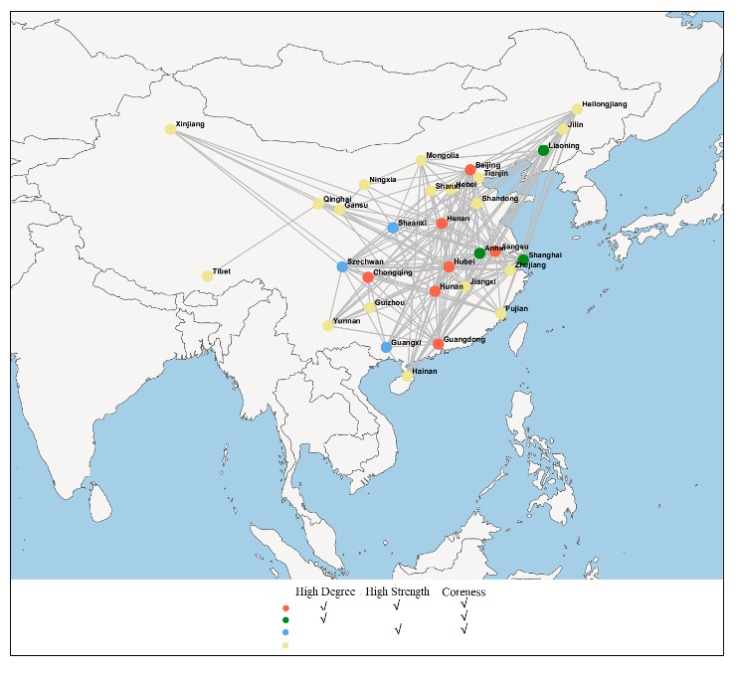
Topological image in geospatial space depicting the network relationship and the centrality of each node over the whole period. The colors of the nodes relate to the intensity of the degree, strength, and coreness.

Taking the COVID-19 epidemic network over the whole period as an example, we calculated the degrees (out-degree and in-degree), strengths (out-strength and in-strength), and corenesses of all the nodes (31 provinces) in Table 3, and we plotted the geospatial network topology map, as shown in Figure 12. It should be emphasized that considering the diversity of the degrees and strengths of each node, the top 10 nodes were defined as having the highest degrees and highest strengths after ranking in descending order. In Figure 12, the color of the nodes indicates the intensities of the three indicators: degree, strength, and coreness. The red nodes correspond to high degree, high strength, and the highest coreness; the green nodes are representative of high degree and the highest coreness; the blue nodes denote high strength and the highest coreness; and the yellow nodes relate to other cases. Concerning the neighboring provinces of the outbreak area of Hubei, three provinces (Hunan, Henan, and Chongqing) show high degrees and strengths as well as the highest coreness. In the same case of the highest coreness, the degree of Anhui Province is high, the strength of Shaanxi Province is high, and neither the degree nor the strength of Jiangxi Province is high. After expanding the considered objects nationwide, due to their high values of degree, strength, and coreness, the provinces of Beijing, Guangdong, and Jiangsu can be regarded as the central nodes of the COVID-19 epidemic network. When analyzing the common characteristics of these three provinces, their high-mobility populations may provide the most reasonable explanation.

**Table 3:** Central properties of 31 provinces during the COVID-19 epidemic. The provinces are arranged in descending order according to the indicators of coreness and degree.

Province	Out-degree	In-degree	Degree	Out-strength	In-strength	Strength	Coreness
Hubei	28	11	39	689	28	717	13
Beijing	19	17	36	46	55	101	13
Henan	17	17	34	33	152	185	13
Guangdong	18	8	26	55	50	105	13
Jiangsu	12	13	25	26	63	89	13
Hunan	13	11	24	42	71	113	13
Liaoning	7	17	24	24	58	82	13
Shanghai	15	7	22	29	17	46	13
Chongqing	11	10	21	28	57	85	13
Szechwan	8	12	20	22	82	104	13
Anhui	9	11	20	26	56	82	13
Yunnan	9	11	20	18	43	61	13
Shandong	7	12	19	12	49	61	13
Heilongjiang	7	11	18	11	47	58	13
Shaanxi	11	7	18	38	49	87	13
Jiangxi	10	7	17	14	20	34	13
Hainan	7	9	16	7	49	56	13
Zhejiang	10	6	16	20	16	36	13
Guangxi	8	7	15	10	80	90	13
Mongolia	8	7	15	14	17	31	13
Hebei	5	9	14	13	43	56	12
Jilin	5	8	13	7	26	33	12
Fujian	7	5	12	10	18	28	12
Tianjin	5	7	12	9	12	21	12
Shanxi	3	7	10	3	22	25	10
Gansu	2	8	10	3	18	21	7
Guizhou	2	5	7	3	9	12	7
Xinjiang	4	2	6	5	2	7	6
Ningxia	0	5	5	0	5	5	5
Qinghai	3	2	5	3	5	8	4
Tibet	0	1	1	0	1	1	1

Some central properties of the 31 provinces during the COVID-19 epidemic are revealed in Table 3. Generally speaking, the maximum coreness of the COVID-19 epidemic network is 13, and provinces with high degrees and strengths tend to have the highest coreness. Furthermore, it can be seen that high strengths and low degrees exist in the provinces of Szechwan, Shaanxi, and Guangxi simultaneously, indicating that these provinces have strong interactions with few provinces. In contrast, the provinces of Liaoning, Anhui, and Shanghai have high degrees and relatively low strengths, which suggests that these provinces have weak interactions with numerous provinces. These findings are more likely to be related to the heterogeneous characteristics of those provinces, such as traffic, population, education, and weather.

**Figure 13:**
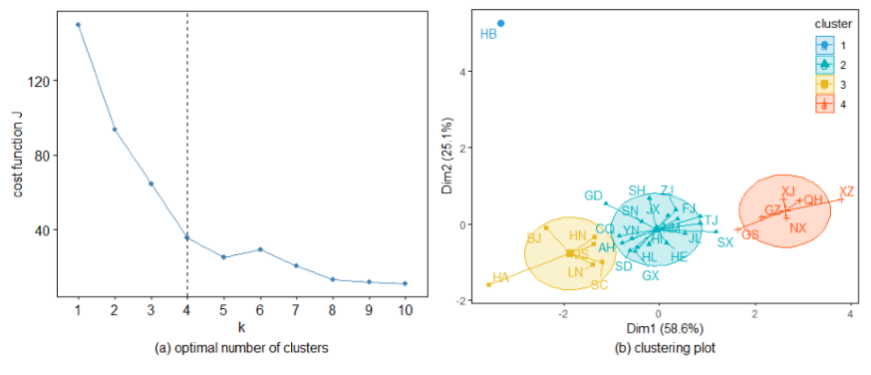
The optimal number of clusters and the clustering plot. The horizontal and vertical axes of the right panel represent the first and second principal components, respectively.

Considering the direction of epidemic transmission, we drew a k-means clustering graph based on the indicators of out-degree, in-degree, out-strength, in-strength, and coreness, as shown in Figure 13. The left panel presents the optimal number of clusters by the Elbow method, and the right panel visualizes the corresponding clusters. We can clearly see that the optimal clustering number of the 31 provinces is 4, and these four clusters obviously exist. Uniquely, Hubei Province formed a single cluster, which can be explained by its role as the initiator of the epidemic. The provinces of Beijing, Henan, Hunan, Jiangsu, Liaoning, and Szechwan are in the same group, and they show relatively high values of the above indicators. In contrast, provinces with lower values, such as Gansu, Guizhou, Ningxia, Qinghai, Tibet, and Xinjiang, belong to another cluster. The remaining provinces are also grouped together. This clustering is similar to that shown in Figure 12 and Table 3, and its uniqueness lies in considering directional factors when investigating the central properties of each province.

The findings in this section comprehensively demonstrate the important nodes in the COVID-19 epidemic network and reveal the transmission path among provinces in mainland China. On this basis, we can further identify some economic and social factors determining the development of this epidemic; finally, effective control can be achieved by imposing some public interventions. With more countries or territories involved in the COVID-19 epidemic, the structure of the world network has become more complex, and it is more urgent to explore the corresponding central properties.

In addition, we attempted to consider directionality in the empirical analysis of the COVID-19 epidemic network, and the results again confirmed the existing findings (see Multimedia Appendix 1).

### Limitations

It is worth mentioning that our study is limited to the transmission of the COVID-19 epidemic in 31 provinces in mainland China, and the k-core decomposition is applicable to unweighted and undirected networks. In the future, more network analysis methods can be considered to explore the epidemic transmission dynamics involving more regions in China or the rest of the world.

### Conclusions

The COVID-19 epidemic is spreading worldwide, and increasing numbers of countries and territories are becoming involved in the network of the outbreak. Identifying the most important nodes and the hierarchical structure of this network has become a priority. Focusing on mainland China, a COVID-19 epidemic network was constructed from the cross-provincial traveling records of confirmed patients; then, three methods, namely degree distribution, k-core decomposition, and influence power, were employed in further structural analysis of the network.

With regard to the empirical results of degree distribution, the power-law distribution suggests that most provinces have either low out-degrees or in-degrees, and only a small fraction of provinces tend to have relatively strong outward or inward transmission effects. In descending order, the three provinces of Hubei, Beijing, and Guangzhou showed the highest out-degrees, and the three highest in-degrees were observed for the provinces of Beijing, Henan, and Liaoning.

The application of k-core decomposition also resulted in some novel findings. First, we verified the hierarchical structure of the COVID-19 epidemic network over the whole period, and more than half of the 31 provinces were found to be in the innermost core. Second, we considered the correlation of the characteristics and coreness of each province, and we identified some significant negative and positive factors. In addition, the variation of the maximum coreness with time was investigated from two perspectives: daily and weekly. An obvious trend of initial rising and subsequent falling appeared both on January 21, 2020, and in the fourth week. To be more specific, considering the dynamic transmission process of the COVID-19 epidemic, the three provinces of Anhui, Beijing, and Guangdong always showed the highest coreness from the third to the sixth week, and Hubei Province maintained the highest coreness until the fifth week but suddenly dropped to the lowest coreness in the sixth week.

Subsequently, the influence power (out-strength and in-strength) was introduced to measure the influence intensity of each k-shell. It was observed that most outgoing and incoming links were from the provinces with the highest corenesses. Moreover, we investigated the dynamic “outgoing” and “incoming” roles of those important provinces, and we found that the out-strength of the innermost nodes was larger than their in-strength before January 27, 2020, after which a reversal occurred.

Our study is committed to making policy recommendations to relevant departments. First, when a public emergency such as an epidemic breaks out, it is necessary to promptly adopt antiepidemic measures (home isolation, wearing masks, etc), and mandatory traffic control should be implemented in a timely fashion to improve the emergency response. Second, measures such as the blockade of Wuhan should not only be implemented in the epicenter of the epidemic, but also in neighboring provinces and territories with high population mobility. The level of the blockade can be determined according to the severity of the local epidemic situation. Finally, in view of the dynamic process of the COVID-19 epidemic, local health organizations should identify the epidemic stage and adjust the controlling measures in a timely fashion.

## Appendix

Multimedia Appendix 1 Supplementary figures and table.

## Abbreviations

GDP: gross domestic product
SARS: sudden acute respiratory syndrome
WHO: World Health Organization
