# Supplementary material for: Analysis of the COVID-19 Epidemic Transmission Network in Mainland China: K-Core Decomposition Study
Source: JMIR Public Health Surveill. 2020 Nov 13;6(4):e24291. doi: 10.2196/24291 (PMC7669363; doi:10.2196/24291)

## **Multimedia Appendix 1** Supplementary figures and table.

**Figure S1.** Correlation diagram of the coreness (in) and characteristics of provinces. The horizontal and vertical axes denote the characteristic variables and coreness (in) respectively. Numbers in the lower right corner of each subgraph are the corresponding correlation coefficient. ** and *** indicate the significance level of 5% and 1%, respectively.


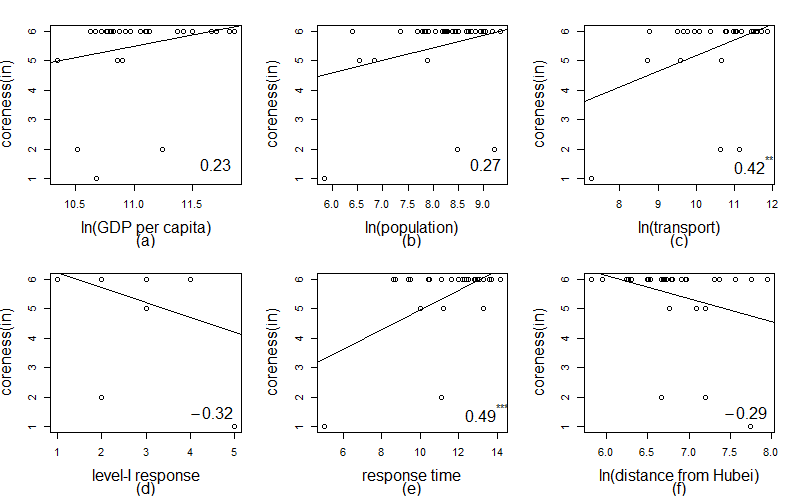


**Figure S2.** Correlation diagram of the coreness (out) and characteristics of provinces. The horizontal and vertical axes denote the characteristic variables and coreness (out) respectively. Numbers in the lower right corner of each subgraph are the corresponding correlation coefficient. ** and *** indicate the significance level of 5% and 1%, respectively.


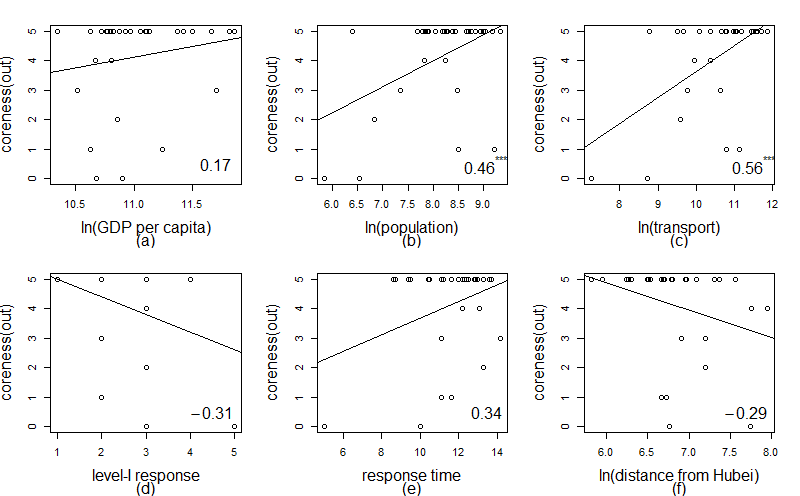


**Figure S3.** Evolution of the maximum coreness, maximum coreness (in) and maximum coreness (out) in the daily COVID-19 epidemic networks. The horizontal and vertical axes correspond to date and the maximum values.


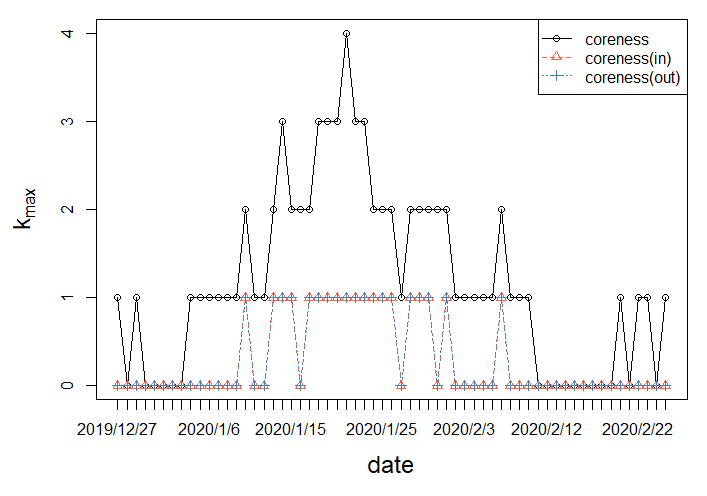


**Figure S4.** Evolution of the maximum coreness, maximum coreness (in) and maximum coreness (out) in the weekly COVID-19 epidemic networks. The horizontal and vertical axes correspond to week and the corresponding maximums.


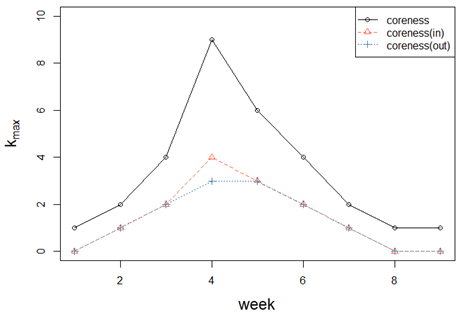


**Table S1.** Coreness, coreness (in) and coreness (out) of 31 provinces of mainland China over the whole period.

| Province | Coreness | Coreness (in) | Coreness (out) |
| --- | --- | --- | --- |
| Anhui | 13 | 6 | 5 |
| Beijing | 13 | 6 | 5 |
| Chongqing | 13 | 6 | 5 |
| Guangdong | 13 | 6 | 5 |
| Guangxi | 13 | 6 | 5 |
| Henan | 13 | 6 | 5 |
| Hubei | 13 | 6 | 5 |
| Hainan | 13 | 6 | 5 |
| Heilongjiang | 13 | 6 | 5 |
| Hunan | 13 | 6 | 5 |
| Jiangsu | 13 | 6 | 5 |
| Jiangxi | 13 | 6 | 5 |
| Liaoning | 13 | 6 | 5 |
| Mongolia | 13 | 6 | 5 |
| Szechwan | 13 | 6 | 5 |
| Shandong | 13 | 6 | 5 |
| Shanghai | 13 | 6 | 5 |
| Shaanxi | 13 | 6 | 5 |
| Yunnan | 13 | 6 | 5 |
| Zhejiang | 13 | 6 | 5 |
| Fujian | 12 | 5 | 5 |
| Hebe | 12 | 6 | 4 |
| Jilin | 12 | 6 | 5 |
| Tianjin | 12 | 6 | 4 |
| Shanxi | 10 | 6 | 3 |
| Gansu | 7 | 6 | 1 |
| Guizhou | 7 | 5 | 2 |
| Xinjiang | 6 | 2 | 3 |
| Ningxia | 5 | 5 | 0 |
| Qinghai | 4 | 2 | 1 |
| Tibet | 1 | 1 | 0 |

**Figure S5.** Dynamic network of COVID-19 epidemic uncovering the node composition and hierarchical structure. The color and label of each node denote the coreness (in) and geographical province respectively. Subgraphs, from top to bottom, from left to right, correspond to 9 weeks in order over the period from December 27, 2019 to February 25, 2020.


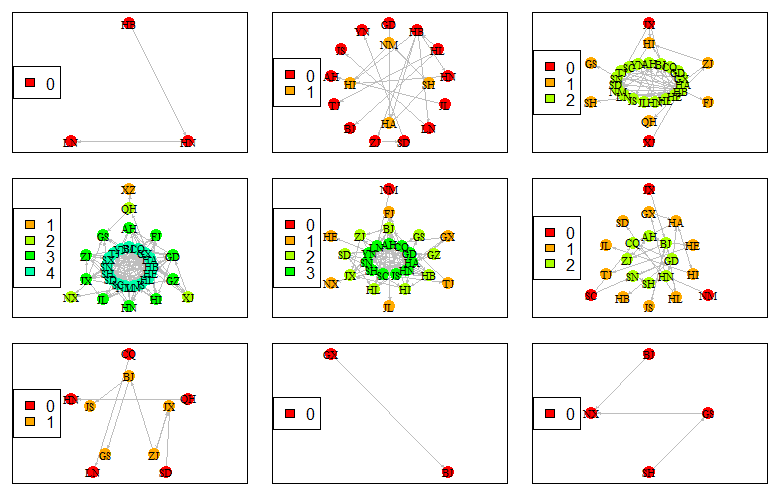


**Figure S6.** Dynamic network of COVID-19 epidemic uncovering the node composition and hierarchical structure. The color and label of each node denote the coreness (out) and geographical province respectively. Subgraphs, from top to bottom, from left to right, correspond to 9 weeks in order over the period from December 27, 2019 to February 25, 2020.


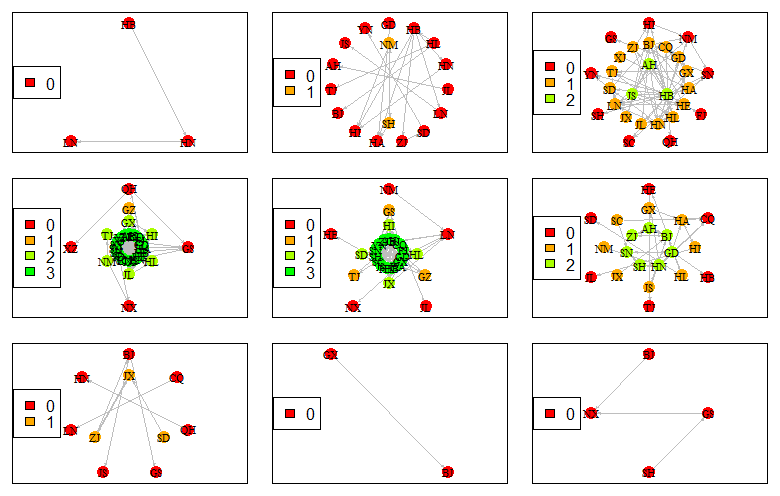

Supplement: Multimedia Appendix 1 [file publichealth_v6i4e24291_app1.docx]
